# Application of bio-orthogonal proteome labeling to cell transplantation and heterochronic parabiosis

**DOI:** 10.1038/s41467-017-00698-y

**Published:** 2017-09-21

**Authors:** Yan Liu, Michael J. Conboy, Melod Mehdipour, Yutong Liu, Thanhtra P. Tran, Aaron Blotnick, Prasanna Rajan, Thalie Cavalcante Santos, Irina M. Conboy

**Affiliations:** 1 Department of Bioengineering, UC Berkeley and QB3 Institutes, Berkeley, CA 94709 USA; 2 Present Address: The 7th affiliated hospital of Sun Yat-Sen University, ShenZhen, Guandong 510275 China

**Keywords:** Proteome, Transgenic organisms, Ageing

## Abstract

Studies of heterochronic parabiosis demonstrated that with age, the composition of the circulatory milieu changes in ways that broadly inhibit tissue regenerative capacity. In addition, local tissue niches have age-specific influences on their resident stem cells. Here we use bio-orthogonal proteome labeling for detecting in vivo proteins present only in transplanted myoblasts, but not in host tissue, and proteins exclusive to one young mouse and transferred during parabiosis to its old partner. We use a transgenic mouse strain that ubiquitously expresses a modified tRNA methionine synthase, metRS, which preferentially incorporates the methionine surrogate azido-nor-leucine (ANL) into newly generated proteins. Using click chemistry and a modified antibody array to detect ANL-labeled proteins, we identify several ‘young’ systemic factors in old regenerating muscle of the heterochronic parabiotic partners. Our approach enables the selective profiling of mammalian proteomes in mixed biological environments such as cell and tissue transplantation, apheresis or parabiosis.

## Abstract (web summary)

Clarifying the source of proteins in mixed biological environments, such as after transplantation or parabiosis, remains a challenge. Here, the authors address this need with a mouse strain that incorporates a methionine derivate into proteins, allowing for their detection using click chemistry and antibody arrays.

## Introduction

The proteome of the cell changes with age and its associated pathologies^1–4^. Proteins produced by the local and systemic environments of organ stem cells broadly regulate the regeneration and maintenance of adult tissues^1–4^. Furthermore, the age-imposed changes in the intensity of evolutionary conserved signaling pathways interfere with productive regeneration of multiple mammalian tissues^3–5^. For example, the age-specific changes in the local and systemic environments of organ stem cells perturb Notch, TGF-beta/BMP, MAPK and Wnt broad-action signaling networks, which regulate the regeneration and maintenance of muscle, brain, liver, blood, etc. tissues^6–8^. Importantly, studies of heterochronic parabiosis (surgical joining of young and old animals) suggest both productive tissue repair and the key signal transduction pathways that control stem cell activation are restored to ‘youth’ in the old parabionts by young systemic factors^9, 10^.

It would be beneficial from academic and clinical stand-points to determine which proteins in tissues of parabiotically connected animals are derived from the circulation of young versus old partner. Such a database of systemic proteins that end up in specific tissues in setting of heterochronic parabiosis, would suggest potentially rejuvenating (young blood) and inhibitory (old blood) molecules with direct effects in a given tissue, also informing on the cross-tissue conservation and differences of the systemic factors from one tissue to another. While biochemical fractionation of serum and plasma can provide some characterization of the molecular differences between young and old circulatory milieu, this technique is fraught with the risk of missing proteins that act in complexes with each other and other macromolecules. In addition, serum and plasma fractionation are indirect approaches based mostly on in vitro studies. And it remains unknown whether or not the age-specific systemic proteins have direct effects in regenerating tissues with their resident stem cells. Candidate factor approaches can be tried, but they require a long time to confirm or rule out just one molecule. In the past decade, these approaches have only yielded a few pro-regenerative molecules, some of which are controversial^11, 12^.

Our approach of choice relies on tRNA synthase that specifically recognizes and incorporates BONCAT(Bio-Orthogonal Non-Canonical Amino acid Tagging) into proteins^13–18^. Specifically, the methionine surrogate azido-nor-leucine (ANL) is incorporated into newly synthesized proteins only in cells expressing this mutant methionine tRNA synthase, MetRS^17^. The mutant MetRS which has a single evolutionary conserved amino acid substitution: 274^L→G^ preferentially incorporates ANL into mammalian cells and in *Drosophila melanogaster* tissues in vivo; and ANL-tagged proteins can be selectively conjugated to dyes or affinity probes and identified^13, 19, 20^.

To facilitate detection by proteomics, we have selected the BONCAT method over the CTAP (cell type-specific labeling with amino acid precursors) where proteomes are tagged with heavy isotope—labeled precursors^16, 21, 22^; and over the incorporation of Met analogs azidohomoalanine (AHA) and homopropargylglycine (HPG), which do not allow one to selectively profile young versus old proteomes in settings of parabiosis^23^.

To advance MetRS^L274G^ ANL labeling technology to live mammals, we have developed and characterized a novel transgenic mouse strain, in which mutant *MetRS*^*L274G*^ is broadly expressed (*MetRS*^*L274G*^ mice). Our data demonstrate the survival and vigor of these animals as well as the effective proteome labeling with ANL of cells in vitro and all examined tissues in vivo. Importantly, ANL tagging using our dosage did not perturb the key properties of the proteins, such as the rejuvenating effects of young ANL labeled serum on the old muscle progenitor cells in vitro or the enhancement of old muscle repair in vivo while still allowing the detection by Click-western, FUNCAT and the bio-orthogonal proteomics profiling.

We have performed transplantation of *MetRS*^*L274G*^ myoblasts into muscle of C57BL/6 mice, which demonstrated a new capability for identification of transplanted cell proteomes without their re-derivation from host tissues. And we have established parabiotic pairings between the young *MetRS*^*L274G*^ mice and old C57BL/6 mice, which yielded data on the candidate systemic rejuvenating factors, e.g., the ‘young’ proteins that have traveled through the parabiotic circulation and were derived from the aged muscle.

## Results

### Characterization of the transgenic mice that broadly express *MetRS*^*L274G*^

**Fig. 1:**
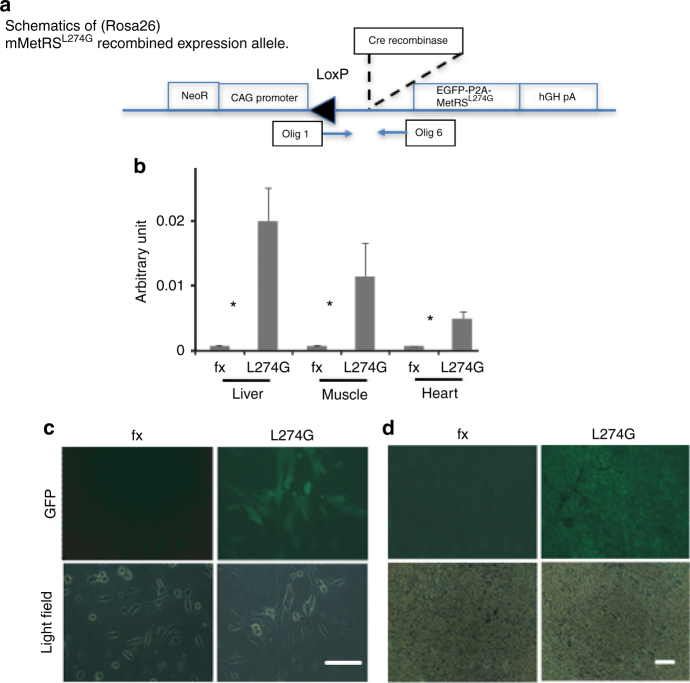
The expression of GFP-tagged MetRS^L274G^ upon ‘Stop signal’ excision. **a** Schematic representation map of the allele after CMV-Cre mediated ‘Stop signal’ excision (MetRS^L274G^ mice). **b** QT PCR demonstrates that transgene expression is indeed induced upon Cre-LoxP mediated excision of the ‘STOP’ signal. *P < 0.05, Wilcoxon rank sum test. s.e.m., *n* = 3 for each genotype. **c** Cells were isolated from indicated genotypes and cultured in F10, 20% FBS for 24 h after which the cells were imaged on bright field and for GFP fluorescence. **d** Muscles from three separate mice were dissected, cryo-sectioned and imaged on bright field and for GFP fluorescence. 3–4 experimental replicates (sections) were assayed for each animal. Representative images are shown. *Scale bar*, 100 μm

To enable the in vivo labeling of mammalian proteome via broad expression of m*MetRS*^*L274G*^, we crossed floxed STOP-EGFP tagged *MetRS*^*L274G*^ mice (i.e., fx mice) with CMV-Cre mice (Fig. 1a). Fx mice were generated using conventional techniques, in which the previously published m*MetRS*^*L274G*^ ^13, 15, 24^ and *EGFP* constructs are inserted into the *Rosa26* locus with Floxed STOP sequence (Supplementary Fig. 1). Breeding pairs of these mice (generously provided by Erin Schuman, Max Planck Institute for Brain Research, Frankfurt, Germany), were genotyped and established into a colony; and animals harboring homozygous fx alleles were crossed with CMV/*Cre* mice (Jackson Labs, *N* > 10 on C57BL/6 background), thereby enabling constitutive broad expression of *mMetRS*^*L274G*^. F1 progeny containing both transgenes Rosa^wt/ex^Cre^CMV^ *MetRS*^*L274G*^ (i.e., *MetRS*^*L274G*^ mice) were identified by polymerase chain reaction (PCR) genotyping (Supplementary Fig. 1) and used for these studies, as well as to establish the founder strain. The PCR, PCR with reverse transcription and direct GFP fluorescence confirmed the excision of the STOP signal and expression of the m*MetRS*^*L274G*^ and *EGFP* in the cells and tissues of these animals (Fig. 1c, d).

In previous studies, mice fed with the methionine homolog AHA had similar body weight as the animals on a regular diet^25^, indicating that the mouse proteome is amenable to tagging with the non-canonical amino acids without toxicity. To confirm these results in our ANL experimental system, we demonstrate that *MetRS*^*L274G*^ mice are viable, fertile, and do not exhibit weight loss as compared to the wild-type mice and fx littermates, both without and with ANL administration (Supplementary Fig. 2).

These results demonstrate successful in vivo expression of the *MetRS*^*L274G*^ and establish that BONCAT does not negatively affect the health, striving and fertility of animals.

### Ubiquitous *MetRS*^*L274G*^ enables broad labeling of mammalian cells and tissues with ANL

**Fig. 2:**
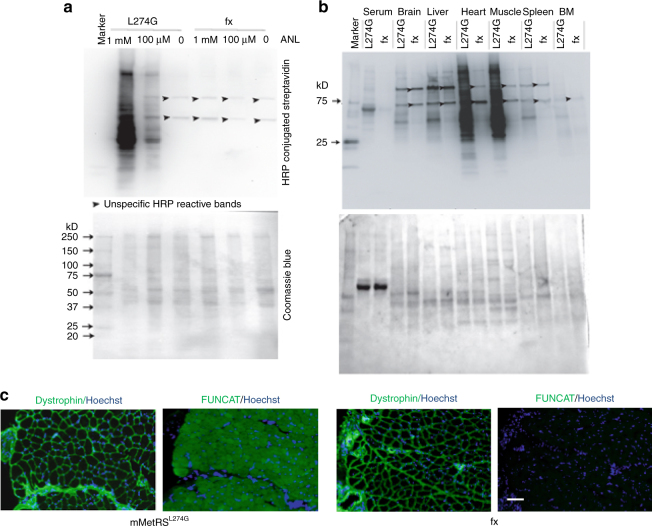
Bio-orthogonal metabolic labeling in mammalian cells in vitro and in mice in vivo. **a** Click-western blotting was performed as described in Methods. The in vitro BONCAT of MetRS^L274G^ fibroblasts has resolved an ANL dose-dependent protein tagging (smears), as compared to the low background of fx cells. The nonspecific bands (*arrowheads*) were present even without ANL treatment in MetRS^L274G^ samples and in fx samples. Coomassie blue image shows similar protein loading for each lane. Similar results were obtained with cells derived from three animals of each strain. **b** In vivo Click-western blotting, which was performed as detailed in Methods, has resolved the incorporation of ANL into all examined tissues of MetRS^L274G^ mice (smears of proteins), as compared to the background signal of fx tissues. The same non-specific bands as described above were present in all samples (arrowheads). Coomassie blue staining is shown as loading control. Three independent experiments with each mouse strain, yielded similar results. **c** 10-micrometre TA muscle cryosections were prepared and assayed by FUNCAT; and the adjacent sections (~ 10 micrometre away) were stained with dystrophin antibody. Robust FUNCAT signal was detected in MetRS^L274G^ dystrophin positive muscle, as compared with the low background fluorescence of fx muscle. *Scale bar*, 100 μm. Three independent experiments with each mouse strain, yielded similar results

To examine the feasibility of *MetRS*^*L274G*^ cells for bio-orthogonal proteome labeling, we isolated neonatal fibroblasts from 1- to 2-day-old *MetRS*^*L274G*^ pups and incubated these cells with ANL in culture. Click reaction and western blotting was used to detect ANL incorporation into proteins^13^. As shown in Fig. 2a, proteome labeling was observed in *MetRS*^*L274G*^ fibroblasts in the ANL dose-dependent manner ranging from 100 micromolar to 1 millimolar. Identical low background – noise was detected in *MetRS*^*L274G*^ cells without ANL treatment and in fx cells that were cultured with 1 mM ANL. The ANL treated *MetRS*^*L274G*^ cells were also eagerly detected by the Clicked AlexaFluor 488 in FUNCAT (fluorescent non-canonical amino acid tagging^26^), as compared to the low background fluorescence of the negative controls (Supplementary Fig. 3A).

We next studied the success of in vivo mammalian BONCAT by administering ANL via intraperitoneal (I.P.) injections to the *MetRS*^*L274G*^ and the negative control fx mice daily for 6 days, after which brain, heart, skeletal muscle, liver, bone marrow and blood serum were isolated. Tissue lysates were prepared and Clicked as described^26^ and the in vivo ANL-labeling of mammalian proteome was assayed by the Click-western and FUNCAT. Click-western blotting resolved robust proteome labeling in all tissues from *MetRS*^*L274G*^ mice as compared to the low background of fx- samples, which was similar to that of in vitro negative controls (Fig. 2b). The highest in vivo incorporation of ANL was detected in heart and skeletal muscle, which fits well with rapid protein turn over in these tissues and their rapid growth in juvenile animals^27–29^. In agreement with the Click-western data, the in vivo incorporation of ANL into proteins of *MetRS*^*L274G*^, tibial﻿is a﻿nterior (TA) mus﻿c﻿le was detected by FUNCAT, well above the background fluorescence of the TA from fx mice that were identically treated with ANL in vivo (Fig. 2c and Supplementary Fig. 4).

These results establish that L274G mutation enables mammalian bio-orthogonal proteome tagging and detection by Click western and fluorescence both in cell culture and in vivo.

### ANL-based detection of *MetRS*^*L274G*^ transplant without re-derivation from host muscle

**Fig. 3:**
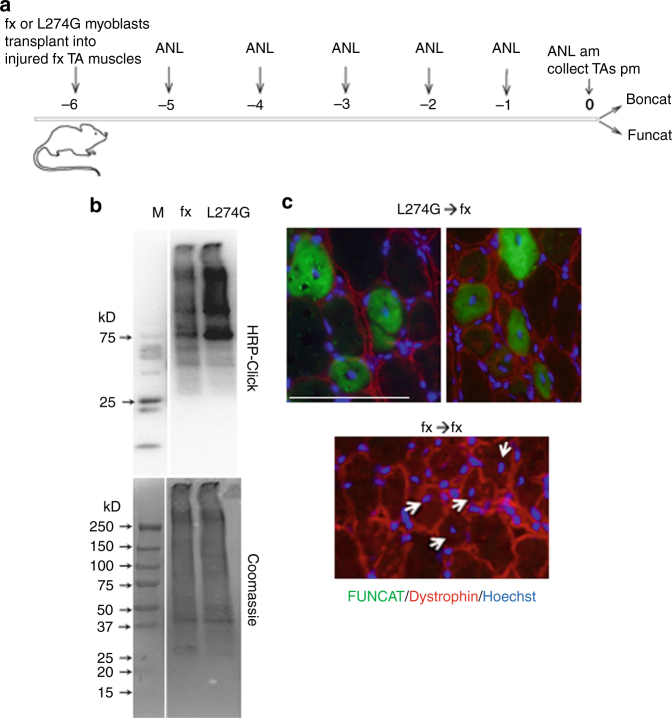
Detection of newly synthesized proteins in fx muscles transplanted with MetRS^L274G^ primary myogenic cells. **a** Scheme of the experimental design. The TA of fx mice were injured with dry ice. Primary myogenic cells derived from MetRS^L274G^ or fx mice were transplanted into the injury sites. ANL was injected for 6 consecutive days I.P. into transplanted animals after which TA muscles were collected. **b** Click-chemistry western blotting on lysates of fx muscles that were transplanted with MetRS^L274G^ versus fx myogenic cells detects ANL-tagged proteins (smearing) in the MetRS^L274G^ samples, as compared to the background signal of fx protein lysates. Coomassie blue staining served as a loading control. Similar results were obtained in three independent experiments. **c** FUNCAT assay in conjunction with anti-dystrophin immunofluorescence and Hoechst nuclear co-stain were performed, as above. The ANL labeling (*green* fluorescence) of dystrophin positive (*red outlines*) myofibers that have been recently regenerated (central Hoechst positive nuclei) was reliably detected in fx TAs that were transplanted with the primary MetRS^L274G^ myoblasts. The negative controls, fx TAs that were transplanted with fx primary myobalsts were FUNCAT negative, while as expected, dystrophin positive and with central nuclei at the sites of transplantation (*white arrowheads*). Similar results were obtained in three independent experiments. *Scale bar*, 100 μm

Encouraged by these findings, we decided to study whether transplanted *MetRS*^*L274G*^ myogenic cells would be detected in host muscles without their re-derivation solely through the in vivo BONCAT. Activated by injury muscle stem cells were isolated from *MetRS*^*L274G*^ and the negative control, fx mice and were cultured for 3–4 weeks, during which time satellite cells give rise to primary myogenic precursors^30, 31^. These myogenic precursors from each strain were transplanted into the fx host muscle following freeze injury^1^, which provides a localized permissive engraftment niche (Fig. 3a). The fx host mice were treated with ANL in vivo for 6 days, as above, after which their TA and Gastroc muscle were isolated and analyzed by Click-western (Fig. 3b) and FUNCAT (Fig. 3c). The de-novo synthesized in vivo ANL-tagged proteins were robustly detectable in fx tissues transplanted with *MetRS*^*L274G*^ myogenic cells, as compared to the low background of fx muscle transplanted with fx cells (Fig. 3). The success of cell transplantation was confirmed by the identification of sites with clusters of newly formed centrally nucleated dystrophin positive myofibers that, as expected, were present in transplants of both genetic backgrounds and were FUNCAT + only in the case *MetRS*^*L274G*^ transplants (Fig. 3c).

These data demonstrate a capability to detect proteomes of transplanted cells in host tissues in vivo without re-derivation and to label de-novo translated proteins at any time after transplantation.

### ANL selectively labels *MetRS*^*L274G*^ proteome in settings of parabiosis with C57BL/6 mice

**Fig. 4:**
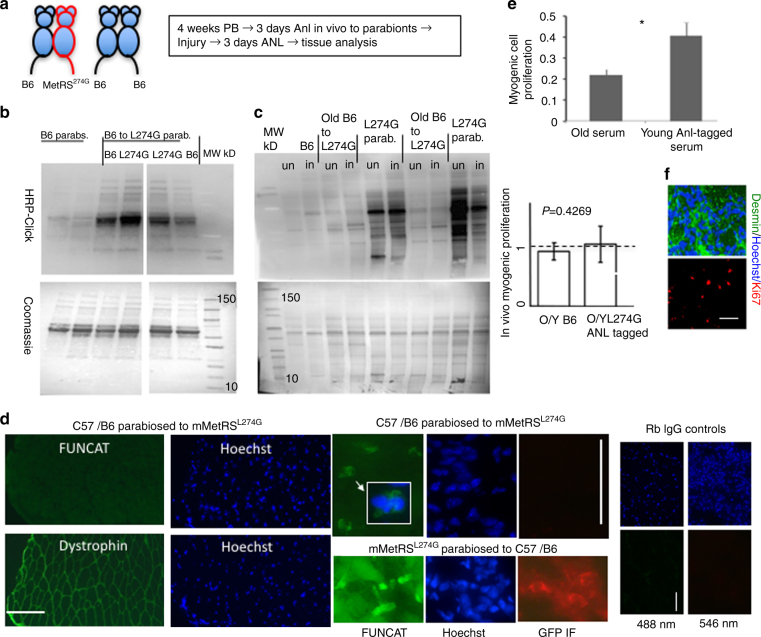
Bio-orthogonal proteome labeling in the setting of heterochronic parabiosis. **a** Schematic representation of the study. **b** Click-western blotting on serum proteins from the heart-bleeds of MetRS^L274G^ parabionts and their C57BL/6 partners and the negative control syngeneic C57BL/6 pairs. **c** Click-western blotting resolved the robust selective in vivo ANL-labeling of the proteomes from uninjured (un) and injured (in) Gastrocnemius muscle of MetRS^L274G^ parabionts (Parabs) and much lower ANL-labeling of proteins that were derived from muscle of their C57BL/6 partners; however, this signal was above the background of the negative control, syngeneic C57BL/6 pairs. Coomassie blue staining demonstrates equal protein loading. Similar results were obtained in at least three independent experiments with each cohort. **d** FUNCAT assay in 10-micrometre muscle cryosections from the indicated parabiotic cohorts has confirmed the selectivity of ANL-labeling of MetRS^L274G^ proteome in settings of parabiosis. A few mono-nucleated cells with FUNCAT signal above that of the nearby tissue were identified in the C57BL/6 muscle (20× image and an enlarged overlay). These FUNCAT + cells were not MetRS^L274G^ leukocytes, as per negative GFP immunostaining (*red*). *Scale bar*, 100 μm. Similar results were obtained in three old C57BL/6 mice independently parabiosed to young MetRS^L274G^ mice and analyzed by FUNCAT. **e** Proliferation rates of old C57BL/6 satellite cells were compared in culture, in presence of serum from young MetRS^L274G^ mice that were administered with ANL in vivo versus the serum from old C57BL/6 mice. Shown are means and SD; *N* = 3, *P* < 0.05 (two-tail Student *t*-test). **f** Muscle regenerative index (the numbers of proliferating Ki67 ^+^ Desmin ^+^ myogenic cells) at 3-days post CTX injury; all parabionts were administered ANL in vivo. Representative immunostaining images are shown in right panels and quantification of the percent in vivo myogenic cell proliferation normalized to the percent myogenic cell proliferation in young muscles that is set at 1 (*dashed line*), is shown in the left panel bar graph. Shown are means and SD. *N* = 3, *P* = 0.426908 (two-tail Student *t*-test). C57BL/6 versus young MetRS^L274G^. *Scale bar*, 100 μm

To pursue our main goal of studying BONCAT in setting of heterochronic parabiosis we have established pairs of young (3–4 month old) *MetRS*^*L274G*^ mice joined with the old (22–24 month old) C57BL/6 mice, using six young-to-old C57BL/6 pairs as controls (Fig. 4a). The success rate for the old C57BL/6 to young MetRS^L274G^ parabiosis was 78% (seven pairs out of nine survived), and the rate for old C57/B6 to young C57/B6 was 46% (6 of 13 pairs survived). The overall success rate was 59%, which is typical for such heterochronic parabiosis studies. Therefore, MetRS^L274G^ transgene does not appear to increase the incidence of parabiotic disease. The pairs were maintained for 5 weeks with daily observation and weighing of the animals, and the parabionts of all genetic backgrounds were healthy, with no detectable adverse reactions, rejection or inflammation (including the *MetRS*^*L274G*^ to old B6 pairs, with or without ANL).

After 4 weeks in parabiosis, ANL was administered to all animals for 6 days at 0.2 mmol/kg. At day 3, one TA and one Gastroc muscle per mouse were injured by cardiotoxin, as published^3, 7, 32^. Three days later injured and uninjured muscle were isolated, and cell-free blood serum and blood cell pellets were collected from heart bleeds.

Click-western was performed, as described above on the parabiotic serum where it resolved the in vivo ANL-tagged proteins in heart bleeds, validating the blood chimerism (Fig. 4b). And the PCR with Cre-specific primers (Supplementary Table 1) confirmed the blood chimerism in blood cells that were derived from the heart bleeds of the *MetRS*^*L274G*^ to C57BL/6 pairs (Supplementary Fig. 5).

After confirmation of the success of parabioses, we have performed the Click-western and FUNCAT on tissues of the old C57BL/6 mice and their young *MetRS*^*L274G*^ parabiotic partners. Click-westerns have revealed the preferential incorporation of the ANL that has been administered to parabionts in vivo into the muscle of *MetRS*^*L274G*^ mice, confirming the selectivity of the proteome labeling by BONCAT in the mixed parabiotic environments (Fig. 4c). And interestingly, some ANL-tagged proteins were consistently detected by Click-westerns in muscle of the C57BL/6 mice sharing blood with the *MetRS*^*L274G*^ parabionts, as compared to the background signal of the syngeneic C57BL/6 pairs (Fig. 4c).

Providing further validation of the data obtained by Click-westerns, FUNCAT demonstrated higher ANL-based protein fluorescence in muscle of the *MetRS*^*L274G*^ parabionts as compared to the muscle of their C57BL/6 partners (Fig. 4d and Supplementary Fig. 3C—FUNCAT and dystrophin immunofluorescence). Interestingly, we detected a few rare cells in the old C57BL/6 muscle with higher FUNCAT signal than the rest of the tissue, suggesting preferential interactions with the circulatory ANL-tagged proteins from young *MetRS*^*L274G*^ parabionts; these were not *MetRS*^*L274G*^- derived cells such as circulating leukocytes, as they were negative for GFP immunofluorescence (Fig. 4d). Muscle of C57BL/6 syngeneic pairs were consistently negative by FUNCAT (Supplementary Fig. 3B).

The intensity of the Click-western and FUNCAT signals in C57BL/6 muscle is limited by the dose and duration of the ANL pulse, and it is expected that with longer labeling and higher ANL dose there will be more ANL-tagged proteins in the parabiotic partners of *MetRS*^*L274G*^ mice. The current regiment of six daily administrations allowed us to see both the selectivity of *MetRS*^*L274G*^ BONCAT in mixed environments, e.g., muscle tissue of parabiotically joined animals, and also to detect the ANL-tagged *MetRS*^*L274G*^ derived proteins that traveled through the shared circulation and rendered some B6 tissues labeled by Click-westerns and FUNCAT.

We also confirmed the phenomenon of association of systemic in vivo ANL-tagged proteins with myogenic cells by incubating cultured primary C57BL/6 myoblasts with serum from the ANL-treated *MetRS*^*L274G*^ mice versus the negative control ANL-treated fx mice, and performing Click-western and FUNCAT (Supplementary Fig. 6).

Notably, we established that the incorporation of ANL in vivo into serum proteins (in *MetRS*^*L274G*^ young mice) did not interfere with their rejuvenating properties. This conclusion is drawn upon the following two observations. In the first experiment, we have assayed the proliferation of old C57BL/6 satellite cells that were cultured with either their own old serum or with the young serum from the in vivo ANL-treated *MetRS*^*L274G*^ mice; the ANL-treated young *MetRS*^*L274G*^ serum maintained the known positive effects of young serum on the proliferation of old satellite cells^4, 30, 33^ (Fig. 4e, *P* < 0.05; two tail Student *t*-test). In the second study, the in vivo numbers of Ki67 ^+^ /Desmin ^+^ myogenic cells were quantified in muscle sections of old C57BL/6 parabionts. Sharing the systemic milieu with either ANL-tagged young *MetRS*^*L274G*^ partner or with the young C57BL/6 partner produced similar enhancement of the old C57BL/6 myogenic cell proliferation^2, 6, 9^ (*P* = 0.426908, two tail Student *t*-test), Fig. 4f.

These results establish an approach for bio-orthogonal proteome tagging in settings of heterochronic parabiosis.

### Modified for bio-orthogonality antibody arrays reveal a number of ‘young’ ANL-tagged proteins that were transferred to old muscle through heterochronic parabiosis

**Fig. 5:**
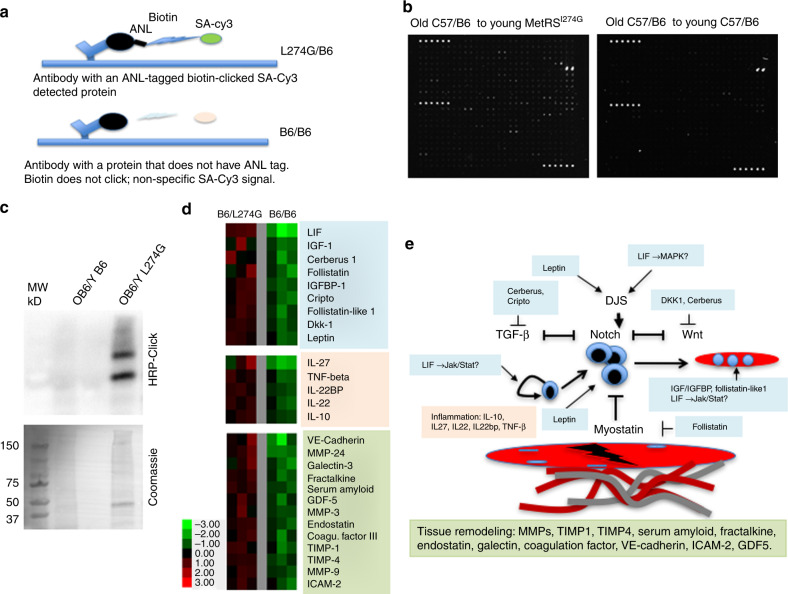
Proteomics of the ANL labeled proteins that are derived from muscle of old C57BL/6 parabiotic partners of young MetRS^L274G^ mice. **a** Scheme of the application of antibody arrays to BONCAT focused proteomics. **b** Representative images of scanned antibody arrays that were performed with the muscle lysates from old C57BL/6 animals parabiosed to young MetRS^L274G^ partners as compared to isogenic C57BL/6 pairs. The lysates were isolated from CTX injured TA and Gastroc muscles at 3 days post injury. **c** For these antibody arrays a Click-western blotting was performed to confirm detectable ANL-labeling in the muscle lysates from old C57BL/6 to young MetRS^L274G^ parabionts, as compared to the background noise of the syngeneic C57BL/6 parabionts. Coomassie blue staining demonstrates equal protein loading. **d** Heat Map of the proteins that were detected at >2.0-fold higher levels in the muscle of old C57BL/6 mice parabiosed to young MetRS^L274G^, as compared to those parabiosed to young C57BL/6 animals with statistical significance of *P* < 0.05 (Wilcoxon rank sum test). *n* = 3. Color-coding: myogenic regulators (*blue*), inflammation regulators (*pink*), tissue remodeling proteins (*green*). **e** Schematic cartoon of the possible roles of the young proteins (colored boxes that correspond to protein grouping in the Heat Map) that were detected in the muscle of old parabiotic partners, grouped by canonical roles in regulating myogenic proliferation and differentiation, TGF-beta/myostatin, Notch and WNT signaling pathways, tissue remodeling and inflammation. Question marks signify putative roles

Finally, to advance the field of systemic aging and rejuvenation by direct profiling of young parabiotic proteome in old tissues, we have developed and used a modified antibody array approach on the samples of muscle from the old C57/B6 to young MetRS^L274G^ versus young C57/B6 parabiosis. All parabionts were labeled with ANL in vivo for 6 days, including the 3 days of recovery from muscle injury induced by Cardiotoxin. In conventional proteomics array samples are labeled with biotin through primary amine and proteins are detected by the binding to their cognate antibodies and biotin-streptavidin-Cy3 fluorescence. We have deliberately omitted the amine-based biotinylation and instead Clicked the biotin-alkyne label to protein samples, postulating that without ANL incorporation the biotin-streptavidin-Cy3 background will be lower than the signal of the ANL-labeled proteins (Fig. 5a). To pursue this proteomics approach, we have used the Ray Biotech arrays, which have 301 features, with duplicates for each antibody specificity.

This strategy was successful, as demonstrated by the representative images where a robust signal over background is evident throughout the slide; and an equally successful hybridization and detection procedure for the experimental (Old C57BL/6 to *MetRS*^*L274G*^) and control (syngeneic C57BL/6) samples is confirmed by the internal positive controls, visible as the rows of bright spots (Fig. 5b). Click-westerns were performed to confirm the ANL-tagging of the proteome in the muscle of old C57BL/6 partners of young *MetRS*^*L274G*^ parabionts, as compared to the background signal of the old C57BL/6 partners of young C57BL/6 parabionts (Fig. 5c).

Applying this strategy, we have resolved 70 ANL-tagged proteins in the regenerating after an injury muscle of old C57BL/6 mice that were parabiotically joined with the young *MetRS*^*L274G*^ partners with statistically significant two-fold signal over the background of the identically treated B6/B6 samples (Supplementary Table 2). Three independent proteomics array experiments with the regenerating muscle from each heterochronic parabiosis cohort produced similar results, emphasizing the robustness of this BONCAT-based high throughput approach.

Further validating our findings and their significance, many of the identified proteins are known to be secreted-systemic; and interestingly, many of these soluble young proteins resolved in the old muscle have myogenic properties (Fig. 5d, e). Of these quite a few are likely to enhance myogenic proliferation^34^, by activating the age-specific determinant Notch signaling^1^ or antagonizing the TGF-beta or Wnt pathways, age-imposed elevation which inhibits old muscle repair^6, 7, 35, 36^, Fig. 5d, e. Follistatin-like1 positively mediates glucose uptake into skeletal muscle^37^, supporting homeostatic health of myofibers, and it also interacts with LIF1 in fat browning, which has multiple health-promoting effects^38^.

Another sub-set of young proteins that have been resolved in old muscles represent tissue remodeling factors, including MMPs and their inhibitors, TIMP proteins, VE-cadherin and VCAM2 that promote cell–cell interactions, GDF5 regulator of re-innervation as well as blood clotting and vascular remodeling factors (Serum amyloid, Fractalkine, Endostatin, Coagulation factor III).

And while some of these ‘young’ parabiotic proteins (directly identified in the old muscle) are well described for their positive and diminished with age muscle-related properties (IGF-1 and Follistatin, for example), others are less characterized in this regard (LIF, Leptin, Cerberus, GDF5, TIMP1, TIMP4).

Finally, another class of ANL-tagged proteins that have been identified in old muscle of C57/B6 mice parabiosed to the young MetRS^L274G^ animals are the leukocyte-specific proteins: Interleukin receptors, chemokines, etc.; these are expected as blood cells travel from the young to old parabionts and are present in skeletal muscle where they contribute to adult myogenesis^4, 39^. Of these, IL27, IL10, IL22, IL22bp and TNF-β regulate tissue inflammation^40^.

## Discussion

This work establishes a selective mammalian in vivo metabolic labeling of proteins in mixed environments (parabiosis and cell transplantation) and introduces a new paradigm for identifying and characterizing key determinants of health-youth versus aging-disease, at the base-line and after experimental treatments. This system does not adversely affect the animal health; and the ANL tagged proteins maintain their functionality. The C57BL/6 background of our CMV-Cre/*MetRS*^*L274G*^ animals enables their broad immuno-compatible use in parabioses, blood exchanges and cell transplantation studies. Furthermore, these studies describe an antibody-array high throughput proteomics approach that is adapted for mammalian in vivo bio-orthogonal translation and requires less starting material than mass spectrometry: micrograms instead of milligrams.

A number of new ramifications in the biomedical arena are enabled by this study. Ubiquitous expression of *MetRS*^*L274G*^ allows approaches where it is important to profile age-specific or disease-imposed, genetic-controlled, etc. changes, be those in global proteomes or in specific candidate protein factors. Not only circulatory, but also local tissue determinants of aging and disease can be more easily profiled: in tissue transplants, one can identify the in vivo proteome of the *MetRS*^*L274G*^ expressing cells and tissues directly, at specific time points and without re-derivation.

The identified young circulatory proteins that were found in muscle of old mice provide the most interesting conclusion of these studies. Not just one or two, but many of these young-blood derived proteins possess rejuvenating pro-myogenic properties, and moreover, they are expected to synergize when such young factors reach the old tissue simultaneously. This suggests that not just one, but many youthful circulatory factors improve the health of old tissues in heterochronic environments, and argue against a ‘one silver bullet’ approach to systemic rejuvenation.

With respect to specific candidates, of a particular interest is LIF-1 that has been shown to enhance the repair of injured muscle, but was not well studied in the context of muscle aging and rejuvenation^41^. LIF-1 is well known to activate the Jak/Stat pathway, which has been shown to reduce symmetric division of myogenic precursors, which would possibly signify return to quiescence, and to promote fusion into myotubes (at the other end of myogenic lineage)^42^. But LIF-1 also activates the MAPK signaling, which can induce the Notch activating ligands Delta and Jagged, promoting myogenic proliferation^43, 44^.

Cripto and Cerberus1 are also interesting, as they act as antagonists of TGF-beta1, which increases with age and inhibits the regeneration of old muscle^7, 32, 45, 46^. With respect to the young derived GDF5 (a TGF-beta family member), it promotes muscle innervation that is known to decline with age^47^. And well-known muscle-specific TGF-beta superfamily protein, myostatin that inhibits muscle stem cell proliferation, might be antagonized by the ‘young’ follistatin^48^. Additionally, Cerberus1 also antagonizes the age-elevated Wnt pathway; and another young-derived Wnt inhibitor, DKK-1, is found in old muscle of old mice parabiosed to the young partners.^6, 49, 50^.

Of note, the ‘young’ proteins, which were transferred to old parabionts have known positive effects on other than skeletal muscle tissues^51^. For instance, inhibitors of TGF-beta and BMP and specifically Fractalkine and Cerberus1, were shown to play a role in brain aging and Parkinson’s Disease^51, 52^, as well as in rheumatoid arthritis^53^. LIF regulates T-cell fate^54^; it also has anti-oxidant properties and is implicated in health of brain, bone, etc. tissues^55, 56^. GDF5 promotes cardiac repair^57^. Follistatin-like1, which was transferred to old mice from young parabionts, is among myokines, which have been implicated in the broad positive effects of exercise on heart, brain, liver etc.^58^. Follistatin-like1 also protects cardiomyocites from experimentally-induced injury and promotes endothelial cell function and tissue re-vascularization^59, 60^.

Finally, leptin that we have identified as one ‘young’ systemic protein that is present in old muscle of parabiotically joined mice, broadly regulates hormonal networks, including those controlling reproduction and metabolism; it has numerous anti-aging effects and leptin or its’ receptor knock-out mice are diabetic, obese and short lived^61, 62^. Interestingly, leptin (that can be produced by many tissues, including skeletal muscle) has been shown to have direct positive effects on myogenic cell proliferation, on the expression of muscle markers, muscle-bone cross-talk and it activates Notch^63, 64^. Of note, leptin interacts with another endocrine hormone—oxytocin in its positive effects on the health of muscle, bone, brain and in reduction of reduces obesity^64–68^. Age-imposed leptin resistance^69^ is a known phenomenon that is linked to frailty; and since oxytocin also declines with age^3^, the alterations of leptin/oxytocin axis might represent a key event in mammalian aging that is rescued by heterochronic parabiosis.

Taken together, we demonstrate a useful approach for in vivo profiling of proteomes that orchestrate the young-healthy versus old-diseased tissue states. The initial proteomic data, obtained by this approach, suggest that a combination of therapeutics would be most effective to enhance resistance to age-imposed and pathological organ attrition.

## Methods

### Animals

All procedures were performed in accordance with the administrative panel of the Office of Laboratory Animal Care. The protocol was approved by the UC Berkeley Animal Care and Use Committee. Mice were anaesthetized with isoflurane drop and euthanized via cervical dislocation. Blood samples were collected by heart puncture. Young (2–4 mo) male C57BL/6 mice and CMV-Cre strain (Jackson labs, #00664, #006054) were purchased from the Jackson Laboratory. Twenty-two-month-old male C57BL/6 mice were purchased from the National Institute on ageing. The CAG-floxed-Stop-eGFP-mMetRS^L274G^ (i.e., fx, Supplementary Fig. 1) breeder pairs (from Erin Shuman, Max Planck Institute for Brain Research, Frankfurt Germany) were genotyped, established as a colony and crossed with CMV-Cre strain. F1 progeny (2–4 mo males) containing both the Cre and recombined and expressed MetRS^L274G^ were used (e.g., MetRS^L274G^ mice).

### Genotyping

Tail clips or blood cells were digested in digestion buffer (100 mM Tris-HCl (pH 8.5), 5mMEDTA, 0.2% SDS, 200 mM NaCl, 100ug/ml proteinase K). Then DNA samples were precipitated with isopropanol and then dissolved in TE buffer. To detect the Cre transgene, PCR was performed using OL2642 and OL2643 (Supplementary Table 1, primers were from Elim Biopharmaceuticals. Inc, Hayward, CA, 94545) in a reaction consisting of 2.5 units *Taq* polymerase (Promega) according to the manufacturer’s protocol. A 450 bp band confirmed the presence of the *Cre* transgene. Control samples negative for *Cre* did not amplify a product. PCR using Oligo 1 and 2 produced a product of ~ 441 bp while oligo 5 and 6 produced a ~ 493 bp, testing the presence of the ‘stop signal’ (Supplementary Fig. 1). Oligos 1 and 6 were used to test the excision of the ‘stop signal’. Oligo 3 and 4 were used to test the presence of eGFP tagged mMetRS^L274G^. Oligos 7 and 8 were used to test the presence of Rosa^wt^ allele. Ctrl1 and Ctrl2 amplified the CD79b wild-type allele producing ~ 330 and ~ 500 bp respectively. The PCR reaction was carried out using the following conditions: 95°/5 min→[95 °C/30 s→ 60 °C /30 s→ 72 °C /30 s]x 30cycles→72°/5 min → 4°/∞. Oligo 3 and 2839reverse were used to amplify a fragment, for which oligo1169reverse was used to sequence the PCR product to confirm the mouse L274G mutation.

### RNA isolation and QT PCR

RNA was isolated using the QIAGEN Rneasy kit (Qiagen, Valencia, CA). RNA concentration was assessed using a NanoDrop spectrophotometer (Thermo Scientific, Wilmington, DE). cDNA was made with Superscript III first strand synthesis kit (Invitrogen 18080-051). QT PCR was carried out using RT2 SYBR® Green FAST Master mixes (Qiagen, cat 330602) according to manufacturer’s protocols. Oligo 3 and 162reverse (Supplementary Table 1) were used to test *mMetRS*^*L274G*^ expression. GAPDH amplified with oligos GAPDH-F and GAPDH-R (Supplementary Table 1) was used as normalization. We use 2^−∆Ct^ as the relative expression unit.

### Identification of ANL-labeled proteins through Click-western blotting

Azidonorleucine (ANL) was purchased from Jena Biosciences (catalog number CLK-AA009, CAS#159610-92-1). ANL was added into the media at indicated concentration or I.P. injected into mice at indicated dose. Protein extraction was carried out by homogenizing samples or cells in RIPA buffer (50mMTris pH7.8, 150 mM NaCl, 0.1%SDS, 0.5% sodium deoxycholate, 1% triton-100, 1 mM PMSF, appropriate proteinase inhibitors) without EDTA + Benzonase (Sigma, ≥ 250 units per ml). Homogenates were incubated for 20 min at room temperature. After incubation for 1 h at 4 °C under constant agitation, lysates were spun down at 3000 g, 5 min. at 4 °C. The resulting supernatants (‘lysates’) were precipitated using 5 volumes of cold acetone, washed with 80% acetone and pellets were air-dried and dissolved in 1%SDS in 50 mMTris˙HCL PH8.0 with 1 mM PMSF freshly added. Protein quantification was performed using Nanodrop and RED 660 protein assay (Cat# 786-676, E-bioscience, St. Louis, MO). Similar amounts of protein samples (~ 20 ug) were then clicked with biotin labeled alkyne according to manufacturer’s protocol (ClickIT biotin-alkyne labeling kit, Life Technologies, Molecular Probes C33372) followed by Western blotting. The Western blots were then incubated with 1:200 dilution of horseradish peroxidase (HRP) conjugated streptavidin and imaged with HRP substrates (Advansta, Western Bright ECL k-12045-D20) following manufacturer’s protocols.

### Visualization of ANL labeled proteins by FUNCAT

Cryosections or cells cultured in chamber slide were fixed with methanol and subsequently 4% paraformaldehyde (in phosphate buffered saline, PBS, 137 mM NaCl, 2.7 mM KCl, 10 mM Na_2_HPO4, 1.8 mM KH_2_PO4, pH7.8). Following fixation, slides were washed three times with PBS. Slides were incubated in 0.1% Triton X-100and washed three times with PBS at room temperature under gentle agitation. Slides were blocked with PBS and 1% serum at 4 °C for 1 h. ANL-labeled proteins were clicked by mixing triazole ligand (THPTA, Sigma-Aldrich, CAS Number 760952-88-3, 3 mM), Alexa Flour 488 Alkyne (Life Technologies, Cat. A-10267, 2uM in dimethylsulfoxide), sodium ascorbate (Sigma-Aldrich, CAS134-03-2, 5 mM), and CuSO4 solution (Sigma-Aldrich, 600 uM) in PBS pH 7.8. After each addition, the solution was vortexed thoroughly. Slides were incubated with the click mixture for 3.5 h at room temperature under gentle agitation. Subsequently, slides were washed twice with PBS-Tween (PBS + 0.05% v/v Tween-20) plus 0.5 mM EDTA solution for ~ 15 min each at room temperature under gentle agitation before incubation with Hoechst DNA staining solution or antibodies following regular immunostaining protocol.

### Immunofluorescence

Muscle sections attached to positively charged frosted glass microscope slides were fixed with 70% ethanol at 4 °C overnight and rinsed with 1× phosphate-buffered saline (PBS) the next day. The sections were then blocked for 30 min in 1% staining buffer (1% calf serum in 1× PBS). Samples were permeabilized with 0.1% Triton X-100 in 1X PBS for 5 min at room temperature and subsequently rinsed with 1% staining buffer 3 times, 5 min per rinse. The sections were incubated with primary antibodies diluted to 0.5–1 μg/ml at 4 °C overnight. The slides were rinsed as stated and then coated with secondary antibodies and Hoechst nuclear DNA stain for 2 h at room temperature in the dark. Samples were rinsed with PBS and mounted with Fluoromount. A Zeiss Axioscope fluorescent microscope was used for imaging. antibodies: rabbit anti-GFP antibody (abcam290,1:5000), rabbit anti-Ki67(ab16667, 1:200), and mouse anti-dystrophin antibody (Santa Cruz Biotechnology 58754,1:200) were used at dilutions according to manufacturer’s instructions. Secondary fluorochrome conjugated antibodies were from Life Technologies: goat anti-rabbit 546 (A11010, 1:2000) and goat anti-mouse 488 (A11029, 1:2000). DNA was stained by Hoechst 33342 from Sigma Aldrich (B2261) 1:500 at 1 μg/mL.

### Preparation and culture of primary cells

Primary myogenic cells were prepared and cultured from muscle samples as previously described^30, 32^. Briefly, harvested muscles underwent enzymatic digestion in DMEM (Mediatech) containing collagenase type II (250U/ml; Sigma-Aldrich), 10 mM HEPES and penicillin/streptomycin antibiotics (500 IU/ml, 0.1 mg/ml; MP Biomedicals) at 37 °C for 20 min under agitation. Fat pads and tendons were removed after a quick wash with PBS and repeated rounds of muscle trituration and sedimentation were performed to purify myofibres. Myofiber suspensions were vortexed for 1 min to release satellite cells from digested fibers, passed through a 40mm cell strainer (BD Biosciences) and plated on Matrigel coated (0.04 ug/ml in PBS) dishes and cultured at 37 °C, 5% CO2 in growth medium (Ham’s F-10 (Mediatech), penicillin/streptomycin antibiotics (500 IU/ml, 0.1 mg/ml; MP Biomedicals), and 20% Bovine Growth Serum (Life Technologies/Hyclone), supplemented with FGF-2 (6 ng/ml)). Neonatal fibroblasts were derived as follows: The pups were euthanized and washed sequentially with sterile PBS, 70% ethanol, and then sterile PBS. The pups were then minced and digested with trypsin-EDTA (Sigma, T4049) for 30 min at 37 °C. The tissue was filtered through a sterile mesh and washed with DMEM (Fisher Scientific, 11330032). The cell pellets were resuspended, cultured and expanded in DMEM with 10%FBS (GE healthcare, SH30396) for two weeks. 70% confluent cells were incubated with ANL at indicated concentrations for 24 h. The primary cells were used soon after their derivation from animals that are routinely checked (found negative) for mycoplasma and other pathogens.

### Parabiosis

Animals were connected surgically in parabiosis as described^9^. Briefly, the pair was prepared for surgery with matching incisions made along the proximal flanks, inner limbs ligated at the elbows and knees, and skin ligated between partners to join the two together and establish anastomosis. Post-operative medication included Baytril antibiotic administered twice daily for the first 3 days, and Meloxicam NSAID administered daily for the first week, as directed by veterinarians; anecdotally, we suggest these may help reduce the incidence of parabiotic disease. The parabioses were not set up all at the same time and the study was done as separate experiments: each pair was individually treated with ANL in vivo and then tissues from individual mice were individually isolated and processed for the BONCAT, FUNCAT, muscle regeneration assays, etc. (with at least three experiments with individual animals and 4–5 experimental replicates within each assay).

### Proteomics

Antibody arrays (Mouse L308 Array; RayBiotech AAM-BLG-1-2, GA, 30092) was used to profile the proteins circulating from young L274G parabionts to old C57/B6 parabionts. The samples were run on three different arrays (each of which was done with the two comparative cohorts), but the samples were different overlapping pools of proteins from individual mice of that cohort (for example, A, B, C for one pool, A, B, D for the second pool, etc.). Each protein was examined in duplicates and the means for each of the 301 proteins were compared between the mutant and the WT results for all proteins; and those found to be elevated by 2 fold in the mutant with *P* > 0.05 were considered to be significant and are reported in Fig. 5 and Supplementary Table 2. Proteins were clicked with biotin labeled alkyne as indicated above and dissolved in 1% SDS 50mMTris-HCl(pH8). Nanodrop was used to quantify the clicked product. The arrays were blocked with blocking solution, incubated with clicked protein (33 micrograms) in PBS(pH8), washed and detected with Cy3 labeled streptavidin, as recommended by the manufacturer. Arrays were scanned using genePix 4000Bscanner (Molecular Devices, Sunnyvale, CA, 94089) at 532 nm. Feature extraction was done using genePixpro6.1 software (Molecular Devices, Sunnyvale, CA, 94089). For each protein, duplicate spots on each array were averaged, local background fluorescence was subtracted and resulting fluorescence signal was normalized by the internal positive controls on the arrays. Average signals from all 6 arrays (3 experiments for positive and negative samples) were set to 1. Log2 transformation was done so that signal value > 0 is shown as red and signal value < 0 is shown as green. Wilcoxon rank sum test was done between Old C57BL/6/Young MetRS^L274G^ group and Old C57BL/6/Young C57BL/6 group. *P*-value = 0.05 and 2-fold Old C57BL/6/Young MetRS^L274G^ signal over Old C57BL/6/Young C57BL/6 background were selected as the cutoffs for the identified ‘young’ proteins that have been resolved in the old muscle in heterochronic parabioses.

### Quantification and statistical analysis

On the basis of the published body of work that generated statistically significant results in similar experimental set-ups and minimizes the unnecessary use of vertebrate animals, a minimum of 10 mice of each genetic background, MetRS^L274G^ and fx, were analyzed for the recombination of the genetic locus, 3 mice for expression and function of MetRS^L274G^, and 10 mice for the viability and vigor of the animals (without and with ANL). A minimum of 3 independent Click-westerns and FUNCAT assays were performed with MetRS^L274G^ and fx cells and tissues with replicates within each assay. 9 and 13 heterochronic parabioses were established for the MetRS^274G^ to C57BL/6 and C57BL/6 to C57BL/6, respectively; with subsequent derivation and analysis of serum, cells and tissues in minimum of 3 independent Click-Chemistry Westerns, FUNCAT assays and Ray Biotech proteomic antibody arrays for each cohort with replicates within each assay. 3 independent myogenic cell transplantation experiments for each genetic background were performed with subsequent multiple analyses by FUNCAT in tissue cryosecitons and by Click-western blotting with replicates within each assay. No data points were excluded from quantification. The data was processed as means and standard deviations. *P* values of <0.05 that were obtained in Student T Test and Wilcoxon rank sum test were considered statistically significant.

### Data availability

The fx and MetRS^L274G^ mouse strains will be shared with all interested researchers after publication of these studies. The authors declare that all other data supporting the findings of this study are available within the paper and its Supplementary Information files, or available from the authors upon reasonable request.

## Electronic supplementary material

Supplementary Information
