# Addendum: Application of bio-orthogonal proteome labeling to cell transplantation and heterochronic parabiosis

**DOI:** 10.1038/s41467-017-02779-4

**Published:** 2018-03-13

**Authors:** Yan Liu, Michael J. Conboy, Melod Mehdipour, Yutong Liu, Thanhtra P. Tran, Aaron Blotnick, Prasanna Rajan, Thalie Cavalcante Santos, Irina M. Conboy

**Affiliations:** 1 Department of Bioengineering, UC Berkeley and QB3 Institutes, Berkeley, CA 94709 USA; 2 Present Address: The 7th affiliated hospital of Sun Yat-Sen University, ShenZhen, Guandong 510275 China

**Keywords:** Proteome, Transgenic organisms, Ageing

Addendum to: *Nature Communications* 10.1038/s41467-017-00698-y, published online 21 September 2017

We would like to make our readers aware of the publication by Alvarez-Castelao et al., which reports the original development of the Floxed MetRS L274G mice for the cell-specific labelling of proteins in vivo and provides an in-depth characterisation of these mice^1^. Breeding pairs of Floxed mice were graciously donated by Erin Schuman and coworkers well in advance of the publication by Alvarez-Castelao et al. Application of this technology to cell transplantation, selective identification of ‘young’ proteins in the setting of heterochronic parabiosis, and array proteomics was developed by the Conboy laboratory.
