# Author Correction: Application of bio-orthogonal proteome labeling to cell transplantation and heterochronic parabiosis

**DOI:** 10.1038/s41467-020-17049-z

**Published:** 2020-07-10

**Authors:** Yan Liu, Michael J. Conboy, Melod Mehdipour, Yutong Liu, Thanhtra P. Tran, Aaron Blotnick, Prasanna Rajan, Thalie Cavalcante Santos, Irina M. Conboy

**Affiliations:** 1 Department of Bioengineering, UC Berkeley and QB3 Institutes, Berkeley, CA 94709 USA; 2 Present Address: The 7th affiliated hospital of Sun Yat-Sen University, ShenZhen, Guandong 510275 China

**Keywords:** Proteome, Transgenic organisms, Ageing

Correction to: *Nature Communications* 10.1038/s41467-017-00698-y, published online 21 September 2017.

In the original version of this Article, the in-figure legend for Fig. 5d contained an error, in which the labels ‘B6/B6’ and ‘B6/L274G’ were inadvertently swapped. This has now been corrected in the PDF and HTML versions of the Article.

