# Supplementary material for: Application of bio-orthogonal proteome labeling to cell transplantation and heterochronic parabiosis
Source: Nat Commun. 2017 Sep 21;8:643. doi: 10.1038/s41467-017-00698-y (PMC5608760; doi:10.1038/s41467-017-00698-y)
Supplement: Supplementary file 1 — Supplementary Information [file 41467_2017_698_MOESM1_ESM.pdf]

Description of Supplementary Files

File Name: Supplementary Information
Description: Supplementary Figures, Supplementary Tables.

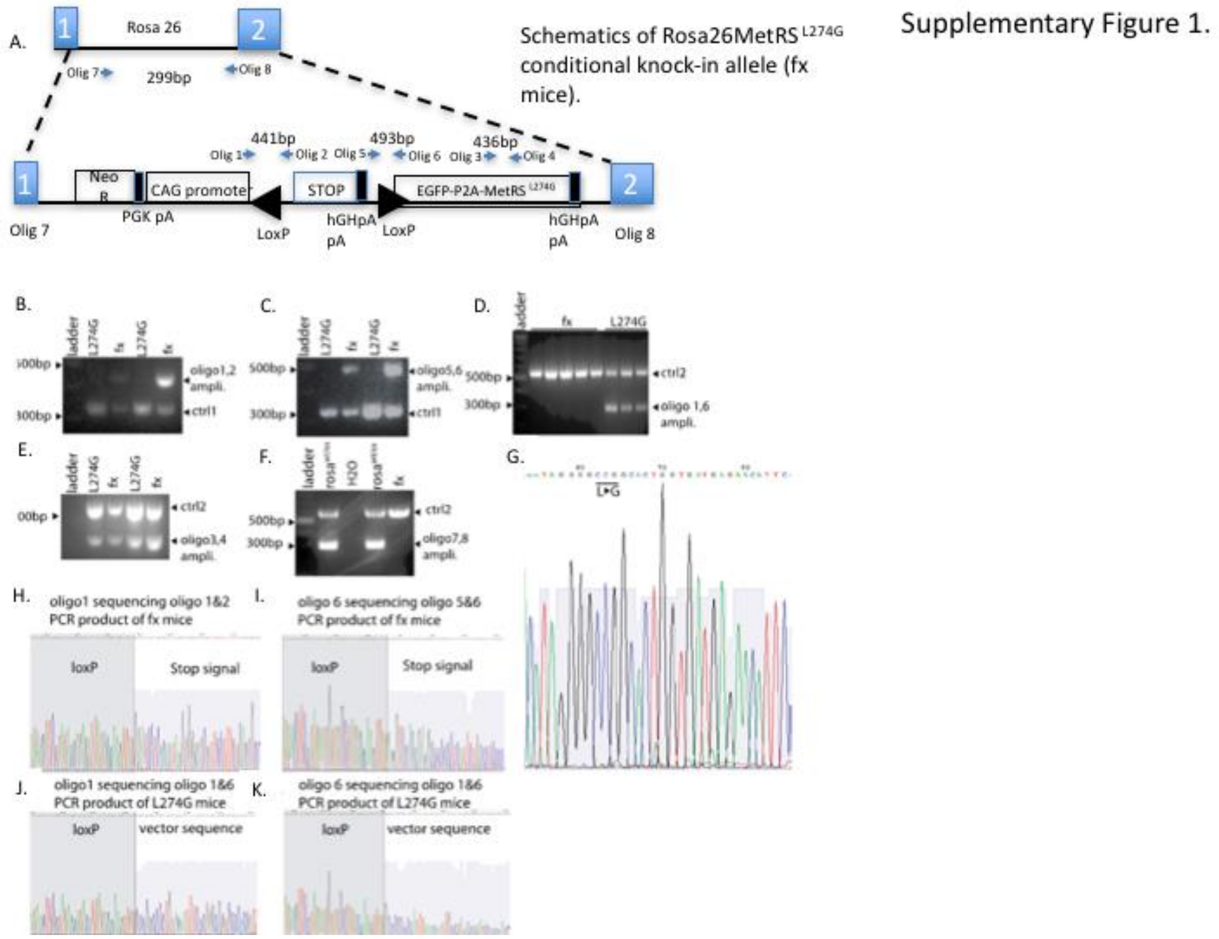

Supplementary Figure 1. The generation of MetRS^{L274G} mice

A. Schematic map of Gt(Rosa26) mMetRS^{L274G} conditional Knock-In allele (fx mice). B-F**:** PCR images showing genotyping. PCR reactions were performed on each studied MetRS^{L274G} animal (>30) and at least on 10 fx mice for genotyping. **G.** Sequencing confirmation of the L274G mutation in MetRS^{L274G} mice. **H-K:** sequencing confirmation of the "stop signal" excision by Cre recombinase. ex, cre recombinase excised allele. fx, conditional knock-in allele. Sequencing confirmation of restriction analysis was done on two MetRS^{L274G} mice and two fx mice.

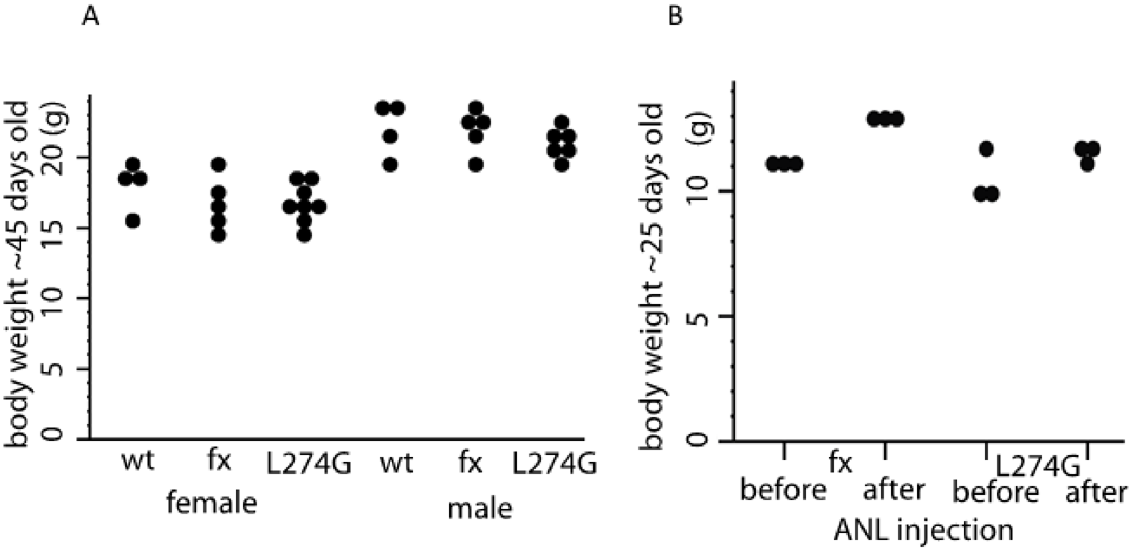

Supplementary Figure 2. Body weight of fx and MetRS^{L274G} mice with and without ANL treatment.

ANL (0.2 mmol/kg) was administered to 25-day-old MetRS^{L274G} mice and fx mice by daily I.P. injections for 6 consecutive days, body weights increased after treatment in both cohorts, consistent with normal growth of mice at this age. By daily observations, fx and *MetRS^{L274G}* mice looked equally healthy and active. A. Body weight of wild type, fx and MetRS^{L274G} mice at the age of ~45 days. B. Body weight of MetRS^{L274G} and fx mice before and after ANL injection that started at the age of ~25 days. Each dot represents an individual animal. No significant differences were detected between MetRS^{L274G} and fx mice (N=3-6, P>0.05, Wilcoxon Rank Sum test). These data were also confirmed by the daily observation of MetRS^{L274G} parabionts.

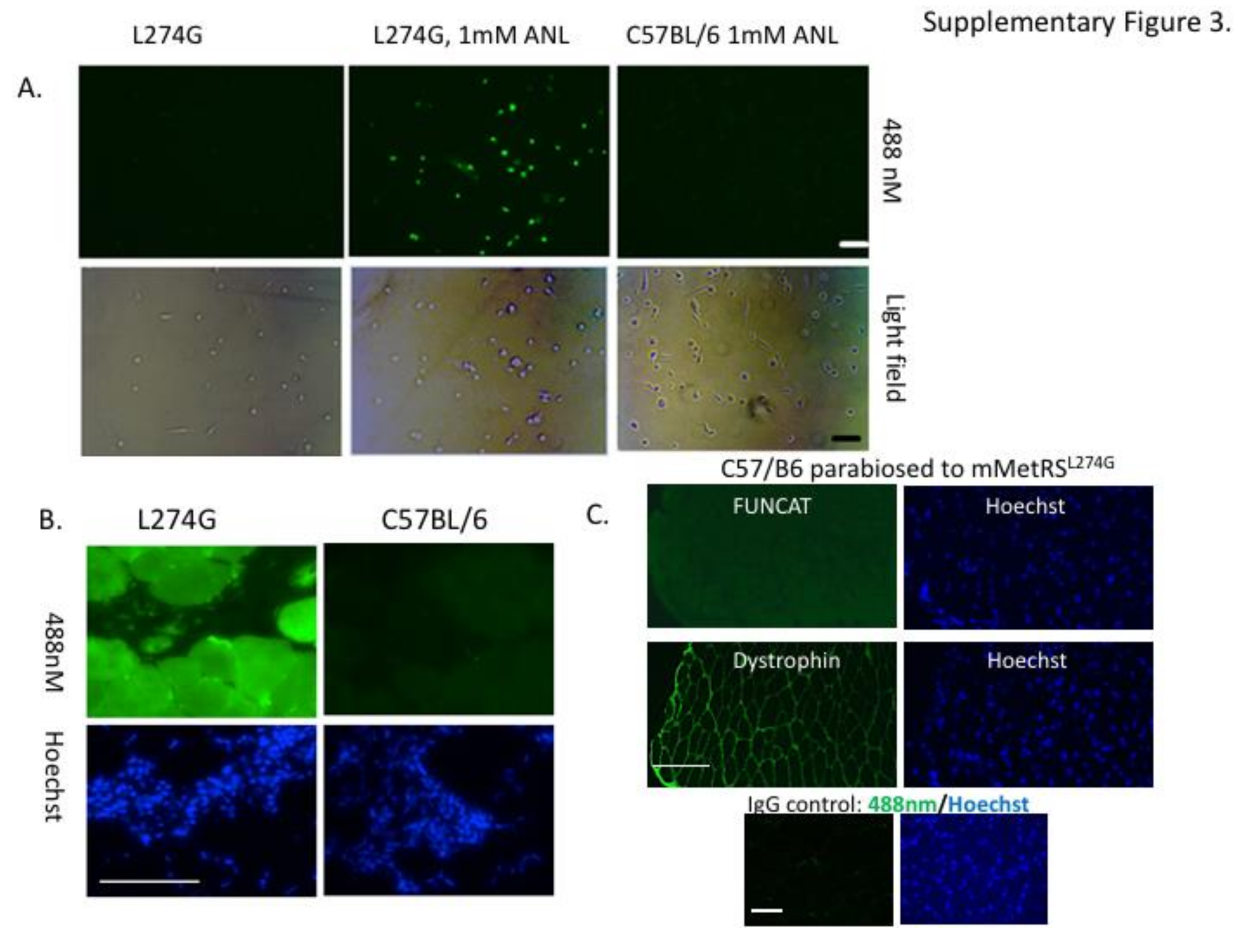

Supplementary Figure 3. Additional FUNCAT studies.

A. FUNCAT on ANL labeled proteins in MetRS^{L274G} cells, as compared to the cells from C57BL/6 mice, treated or not with ANL. Similar results were obtained with at least three independently derived primary cell preparations from three of each: MetRS^{L274G} mice and C57BL/6 mice. **B.** FUNCAT on ANL labeled proteins in muscle cryosections from parabiotic MetRS^{L274G} or C57BL/6 mice that were administered with ANL *in vivo* (as in Figure 4A). **C.** FUCNAT assay and dystrophin immunofluorescence were performed on ~10-micron adjacent sections of C57BL/6 muscle that was derived from parabiotic partners of MetRS^{L274G} animals (with ANL administration *in vivo* as described schematically in Figure 4A). Isotype matched IgG controls for dystrophin immuno-detection are also shown. Hoechst (blue) labels all nuclei. Scale

bar, 100μm. Similar results were obtained from at least three C57BL/6 parabiosed to MetRS^{L274G} mice and three MetRS^{L274G} parabiosed to C57BL/6 mice.

Supplementary Figure 4.

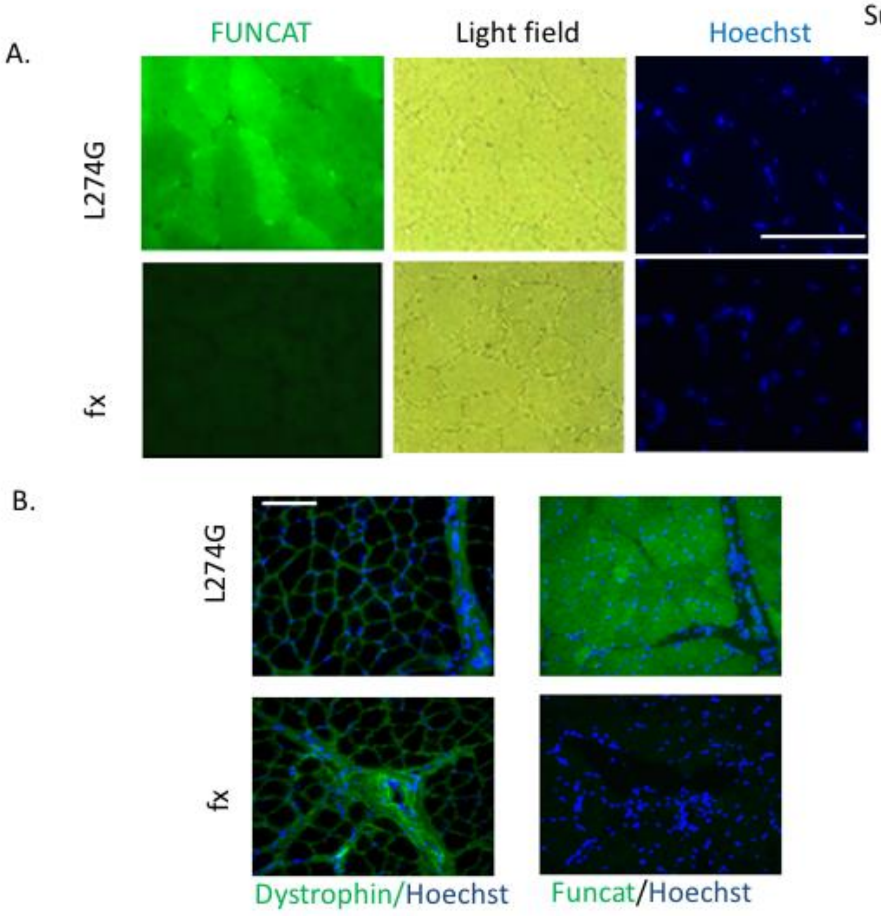

Supplementary Figure 4. FUNCAT comparison between MetRS^{L274G} and fx muscle tissue and **co-detection with dystrophin immunofluorescence.**

Representative images of 10-micron muscle cryosections that were derived from MetRS^{L274G} or fx mice administered with ANL *in vivo*, and assayed by **(A).** FUNCAT or **B.** FUNCAT with dystrophin immunostaining on adjacent sections. Hoechst (blue) labels all nuclei. Scale bar, 100μm. Similar results were obtained with at least 3 MetRS^{L274G} and fx mice.

Supplementary Figure 5.

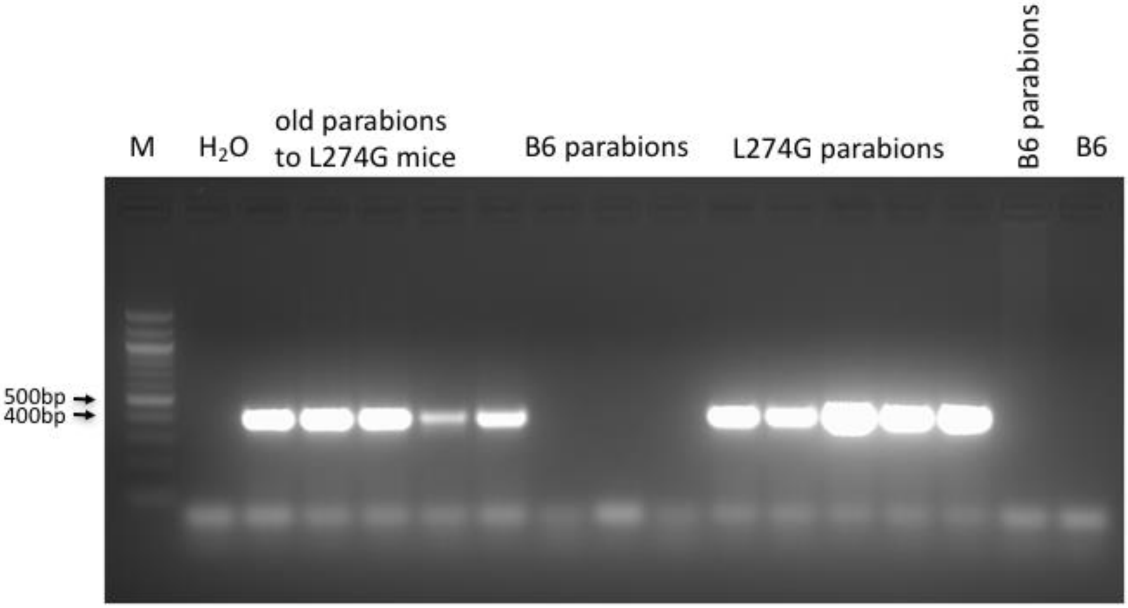

Supplementary Figure 5. Blood chimerism.

Gel electrophoresis on PCR wiith Cre-specific primers that was performed on genomic DNA isolated from heart-bleed derived blood cells of parabionts and control C57BL/6 mice, as indicated. Cre-specific primers (OL2642 and OL 2647) were used to amplify the 450bp Cre DNA fragment. I kb ladder (M). Similar results were obtained from 7 pairs of old C57BL/6 to young MetRS^{L274G} parabionts and 6 pairs of old C57BL/6 to young C57BL/6 parabionts.

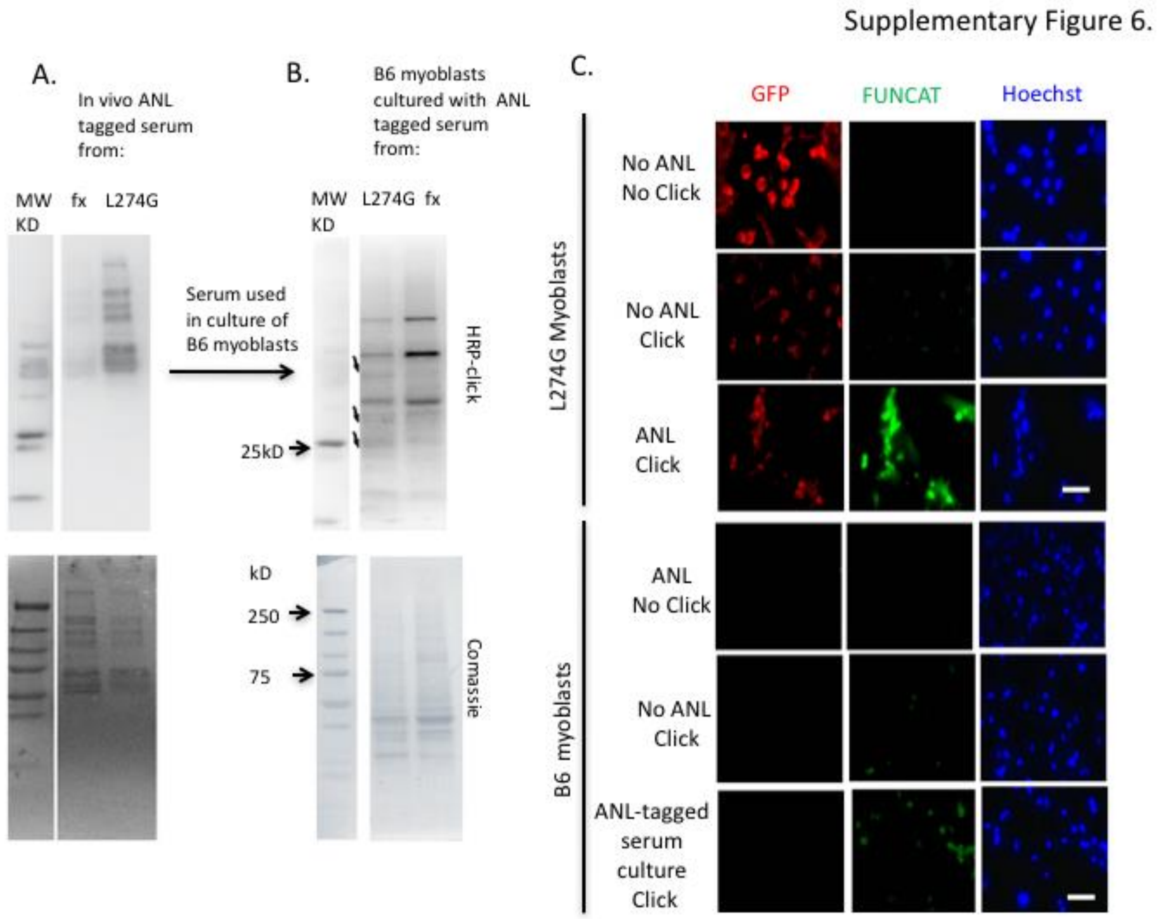

Supplementary Figure 6. Detection of ANL-tagged proteins from primary myoblasts incubated with *in vivo* ANL labeled serum.

MetRS^{L274G} mice and the negative control fx mice were labeled with ANL for 5 days *in vivo*, as in Methods. Their blood serum was collected. A. Click-Chemistry Western Blotting was used to assay for effective ANL tagging of serum proteome in MetRS^{L274G} but not fx mice. B. Primary C57BL/6 myoblasts were cultured with serum from MetRS^{L274G} and fx mice (as illustrated in the schematic) and Click-Chemistry Western Blotting was used to assay for the association of the *in vivo* ANL tagged serum proteins with the C57BL/6 myoblasts in culture. A few above-background (of the fx negative control) bands were detected (arrow heads) C. FUNCAT assay has also detected the above background ANL labeled proteins in C57BL/6 myoblasts that were cultured with MetRS^{L274G} ANL-tagged serum, which rendered these cells visible by the Click-

fluorescence. At least 6 individual serum samples from each: 3 $MetRS^{L274G}$ mice and 3 fx mice produced similar results after incubation with primary myoblasts and subsequent analysis by Click-Western.

Supplementary Table 1: Primers that were used in these studies.

Oligo1	TGGCAGGCTTGAGATCTGG
Oligo2	TTATTGATCCGCGCCTGG
Oligo3	GACCACTACCAGCAGAACACC
Oligo4	AGAAGAGGTAGTTGCCACTATCC
Oligo5	ACGTCCAGACACAGCATAGG
Oligo6	GGACACGCTGAACTTGTGG
Oligo7	CTCTTCCCTCGTGATCTGCAACTCC
Oligo8	CATGTCTTTAATCTACCTCGATGG
Ctrl1forward	GTGGCACGGAACTTCTAGTC
Ctrl1reverse	CTTGTCAAGTAGCAGGAAGA
Ctrl2forward	GAGACTCTGGCTACTCATCC
Ctrl2reverse	CCTTCAGCAAGAGCTGGGGAC
OL2642	tgcctgcattaccggtcgatgc
OL2643	ccatgagtgaacgaacctggtcg
2839reverse	GCTTGGTCACCTCATCCGTC
1169reverse	ACTGGCGAAGGCGACAATAC
162 reverse	CATGCCGAGAGTGATCCCG
GAPDH-F	CCACTTGAAGGGTGGAGCCA
GAPDH-R	TCATGGATGACCTTGGCCAG

Supplementary Table 2: Genes that have been found by the BONCAT focused Antibody array studies. N=3 independent array experiments for each parabiotic cohort. P<0.05 (Wilcoxon rank sum test), 2-fold increase over negative control.

Name	Unigene ID
1. B7-1/CD80	Mm.89474
2. BCMA / TNFRSF17	Mm.12935
3. BLC	Mm.10116
4. CCR6	Mm.8007
5. Cerberus 1	Mm.6780
6. Coagulation Factor III / Tissue Factor	Mm.273188
7. Cripto	Mm.5090
8. DKK-1	Mm.214717
9. Dtk	Mm.424496
10. EGF R	Mm.420648
11. Endostatin	Mm.4352
12. FGF R4	Mm.276715
13. FLRG (Follistatin)	Mm.251710
14. Follistatin-like 1	Mm.182434
15. Fractalkine	Mm.103711
16. Galectin-3	Mm.248615
17. GDF-5	Mm.4744
18. GFR alpha-4 / GDNF R alpha-4	Mm.198399
19. Granzyme B	Mm.14874
20. ICAM-2 / CD102	Mm.394
21. IFN-alpha / beta R2	Mm.6834
22. IGFBP-1	Mm.21300
23. IGFBP-3	Mm.29254
24. IGF-I	Mm.268521
25. IL-10	Mm.874
26. IL-10 R alpha	Mm.379327
27. IL-15 R alpha	Mm.200196
28. IL-17BR	Mm.269363
29. IL-17R	Mm.4481
30. IL-21 R	Mm.155643
31. IL-22	Mm.103585
32. IL-22BP	Mm.331979
33. IL-27	Mm.222632
34. IL-31 RA	Mm.380801
35. IL-9 R	Mm.384

36. KC	Mm.21013
37. LEPTIN(OB)	Mm.277072
38. LIF	Mm.4964
39. MIG	Mm.766
40. MIP-3 alpha	Mm.116739
41. MIP-3 beta	Mm.426373.
42. MMP-24 / MT5-MMP	Mm.389325
43. MMP-3	Mm.4993.
44. MMP-9	Mm.4406
45. PDGF R beta	Mm.4146
46. Progranulin	Mm.1568.
47. Resistin	Mm.1181
48. Serum Amyloid A1	Mm.148800
49. SLPI	Mm.371583
50. SPARC	Mm.291442
51. TARC	Mm.41988
52. TCA-3	Mm.1283
53. TFPI	Mm.124316
54. TGF-beta 1	Mm.248380
55. TGF-beta RI / ALK-5	Mm.197552
56. TGF-beta RII	Mm.172346
57. Thymus Chemokine-1	Mm.293614
58. TIMP-1	Mm.8245
59. TIMP-4	Mm.255607
60. TL1A / TNFSF15	Mm.208152
61. TLR1	Mm.273024
62. TLR3	Mm.33874
63. TMEFF1 / Tomoregulin-1	Mm.422686
64. TNF RII	Mm.235328
65. TNF-beta / TNFSF1B	Mm.87787
66. TRAIL R2 / TNFRSF10B	Mm.193430
67. TRANCE / TNFSF11	Mm.249221
68. TREM-1	Mm.248352
69. VE-Cadherin	Mm.21767
70. VEGF R3	Mm.3291
